# Exploring the link between *Mycobacterium avium* subsp. *Paratuberculosis* and colorectal cancer development

**DOI:** 10.1186/s12876-025-04597-1

**Published:** 2026-01-24

**Authors:** Fatemeh Rangi Tehrani, Negar Asgari, Ailar Jamalli, Ezzat Allah Ghaemi, Taghi Amiriani, Samin Zamani

**Affiliations:** 1 Department of Microbiology, School of Medicine, Golestan University of Medical Sciences, Gorgan, Iran; 2 Gastroenterology and Hepatology Research Center, Golestan University of Medical Sciences, Gorgan, Iran; 3 Golestan Research Center of Gastroenterology and Hepatology, GolestanUniversity of Medical Sciences, Gorgan, Iran

**Keywords:** *Mycobacterium avium* subsp. *Paratuberculosis*, Colorectal cancer, Inflammatory bowel diseases, Polymerase chain reaction, Nested PCR

## Abstract

**Background:**

Colorectal cancer (CRC) ranks third in incidence and fourth in cancer-related mortality worldwide. It typically begins as benign polyps that may progress to malignancy through cumulative genetic alterations. Inflammatory bowel disease (IBD) is a major risk factor for CRC, and Mycobacterium avium subspecies paratuberculosis (MAP) has been implicated in its pathogenesis and may also contribute to CRC development, particularly in regions with MAP-infected livestock.

**Methods:**

In this case-control study which included 147 participants, we analyzed 74 patients with colorectal conditions, encompassing both precancerous and cancerous types, alongside 73 healthy controls (HCs). The Participants underwent colonoscopy at a clinical center in Golestan province, and provided informed consent and relevant health data. Exclusion criteria included recent antibiotic use and a history of gastrointestinal disease. Genomic DNA was extracted from intestinal mucus samples and analyzed using nested PCR.

**Results:**

In our study, the prevalence of MAP was significantly higher in precancerous (46.7%) and cancerous patients (57.1%) compared to HCs (13.3%, *p* < 0.0001), suggesting a possible association with disease progression that warrants further investigation.

**Conclusion:**

The study highlights a strong association between MAP and colorectal neoplasia, especially in patients with precancerous and cancerous conditions. Despite no notable differences in lifestyle factors between groups, MAP’s prevalence points to its potential influence on CRC progression. This warrants further investigation into MAP’s role in CRC, particularly in regions with MAP-infected livestock, for public health insights and prevention strategies.

## Introduction

Colorectal cancer (CRC) is the third most frequently diagnosed form of cancer and the fourth leading cause of cancer-related death, after lung, liver, and stomach cancer. Each year, the number of new CRC cases reported ranges from one to two million. Based on sex-specific data, CRC is the second most prevalent cancer in women and men, with rates of 9.2% and 10%, respectively. It is most commonly found in those aged 50 and above [1]. CRC often begins as a benign polyp, a small clump of cells that forms on the lining of the colon or rectum [2]. In the context of early-onset CRC, four key symptoms that may necessitate medical consultation are persistent abdominal discomfort, blood in the stool, chronic diarrhea, and iron-deficiency anemia. Exhibiting any combination of these symptoms correlates with an increased probability of receiving a colorectal cancer diagnosis [3]. With the passage of time, certain polyps may evolve into cancerous lesions as a result of cumulative genetic changes. These polyps, which are initially harmless, can undergo mutations that trigger uncontrolled cellular growth, ultimately leading to CRC. This ongoing process of cell division and genetic mutation allows these cells to infiltrate the intestinal wall and spread to other areas of the body, such as lymph nodes and distant organs, in a process known as metastasis. Among the various types of polyps, adenomatous polyps and sessile serrated polyps (SSPs) stand out as the two main kinds with the potential to develop into CRC, each with its own specific risk of turning malignant [4, 5]. The onset of colorectal cancer is associated with a variety of factors. Research has shown that individuals with a history of cholecystectomy, diabetes mellitus, inflammatory bowel disease (IBD), or a family history of cancer are at an increased risk for developing this type of cancer. Lifestyle choices play a crucial role as well; risk factors include obesity, physical inactivity, tobacco use, excessive alcohol consumption, and poor dietary habits, such as high consumption of red and processed meats and low intake of fiber, fruits, vegetables, calcium, and other nutrients [6]. Additionally, the likelihood of developing CRC is influenced by age, sex, socioeconomic status, and the composition of the gut microbiome [7]. IBD, which includes Crohn’s disease (CD) and ulcerative colitis (UC), is the third most significant risk factor for CRC [8]. The precise origins of IBD are not fully understood, but it is thought to result from a multifaceted interaction of immunological, genetic, and environmental influences, with infectious organisms such as bacteria being acknowledged as possible factors [9, 10]. In this context, *Mycobacterium avium* subspecies *paratuberculosis* (MAP), traditionally linked to CD and now also implicated in UC, is known to cause Johne’s disease, a persistent intestinal ailment in ruminants [11]. This connection underscores the potential role of MAP in the pathogenesis of both major types of idiopathic inflammatory bowel disease (IBD) [12, 13]. it may also play a role in the CRC cases that often arise as a complication of IBD. Furthermore, in regions where MAP infection among domestic animals is prevalent and environmental contamination with MAP is widespread, it could be implicated as one of the causes of CRC even in individuals who do not have IBD [14, 15]. Research indicates that MAP has the ability to infect intestinal goblet cells, potentially causing both acute and chronic increases in these cells, a condition known as goblet cell hyperplasia. This hyperplasia, which may manifest as transitional mucosa or aberrant crypt foci, represents an early and often overlooked pathological change that can precede sporadic CRC. It is hypothesized that MAP’s invasion of goblet cells could be the initial pathological event in both IBD and sporadic CRC. The persistent infection of the intestinal lining by MAP might be a contributing factor in the progression to CRC [16]. The presence of MAP in the intestinal mucosa may initiate a sequence of inflammatory responses, potentially increasing the risk of developing CRC. Chronic inflammation is acknowledged as a significant risk factor for various cancers, including CRC. This is attributed to its capacity to inflict DNA damage, foster angiogenesis, and facilitate the survival and expansion of cancerous cells [7]. MAP infection may induce an inflammatory environment, potentially disturbing the equilibrium of the gut microbiota. Such a disruption could create conditions that are conducive to the initiation of cancerous processes [17]. Furthermore, the cell wall constituents of MAP, such as lipoproteins and glycolipids, have a role in modulating the host’s immune response. These elements function as pathogen-associated molecular patterns (PAMPs) and are recognized by pattern recognition receptors (PRRs) on host cells. This recognition triggers innate immune responses that may result in chronic inflammation and contribute to the process of tumor formation [18].

The objective of this research is to evaluate the prevalence of MAP in tissue specimens from individuals diagnosed with CRC and those exhibiting precancerous lesions, in comparison with samples from healthy subjects. This investigation seeks to explore the potential association between MAP infection and tumorigenesis. By expanding the scope of analysis to include various stages of disease progression, the study aims to provide a more comprehensive understanding of MAP’s role at different points in the carcinogenic pathway, from early cellular changes to full-blown malignancy. This could offer valuable insights into the mechanisms by which MAP may contribute to the onset and advancement of CRC.

## Materials and methods

### Subjects

In this case-control study, 147 individuals were enrolled, divided into two distinct groups. The first group consisted of 74 patients with colorectal conditions, including 60 with precancerous lesions and 14 with CRC, accounting for 50.3% of the total participants. Precancerous lesions were defined as colorectal polyps or mucosal lesions with histopathologic features consistent with an increased risk of progression to carcinoma, including conventional adenomatous polyps (tubular, tubulovillous, or villous) and serrated lesions with dysplasia. In our cohort, the majority of precancerous lesions were conventional adenomas, with a smaller proportion of serrated lesions.

The second group comprised 73 healthy controls (HCs), making up 49.7% of the participants. All individuals were referred to the colonoscopy center at the Golestan province gastroenterology clinic. Each participant provided informed consent before completing a detailed questionnaire designed to collect relevant health information for the study.

The patient group was selected based on the presence of colon cancer symptoms or verified precancerous and cancerous lesions, as diagnosed through recent medical examinations, colonoscopies, and pathological evaluations as per the gastroenterologist’s assessment. The control group includes individuals devoid of any gastrointestinal cancer history, precancerous lesions, or chronic gastrointestinal conditions. The study’s exclusion criteria encompass recent antibiotic, prebiotic, or probiotic use within the past three months, any invasive medical procedures within the same timeframe, a history of any cancer or infectious or inflammatory bowel diseases, and other intestinal disorders such as CD, IBS, CRC, IBD, UC, or a high predisposition to CRC including hereditary cancer syndromes such as familial adenomatous polyposis.

### DNA extraction

During the colonoscopic procedure, two samples of intestinal mucus were collected from each study participant. Two mucus samples were collected from each participant according to a standardized protocol, sampling the ascending colon and sigmoid colon as predefined anatomical locations. One of these samples was immediately placed on ice within a sterile microtube containing phosphate-buffered saline (PBS) at a pH of 7.4, ensuring preservation during transport to the laboratory for further analysis. Upon arrival at the laboratory, genomic DNA was extracted from the colonic mucosal samples. This process was performed utilizing a DNA extraction kit provided by Favorgen Biotech Corporation, Taiwan, following the protocol specified for gram-positive bacteria. This methodological approach ensures the integrity of the DNA for subsequent analyses.

### Nested-PCR

We used a nested-PCR assay targeting the IS900 element of MAP, following the protocol described by Bull et al. [19] (Table 1), employing two sets of primers (L/AV) previously validated for specificity. All PCR steps were performed in physically separated areas for DNA extraction, reaction setup, and post-amplification analysis to minimize contamination. Each run included a no-template negative control and a positive control consisting of DNA from a reference MAP strain. Only bands of the expected size were interpreted as positive, allowing us to reliably investigate the presence of bacterial DNA across all groups.

**Table 1:** Internal and external primers

Number	Gene	Primer	Amplicon size (bp)	Reference
1	L	F: CTTTCTTGAAGGGTGTTCGG	402	(Bull et al., 2003) [19]
		R: ACGTGACCTCGCCTCCAT		
2	AV	F: ATGTGGTTGCTGTGTTGGATGG	298	
		R: CCGCCGCAATCAACTCCAG		

The target sequence’s amplification was performed in a 14.8 µL reaction mixture, containing 0.75 µL of each forward and reverse primer, 5 µL of master mix, 2 µL of DNA extracted from each specimen, and 9.3 µL of DEPC-treated water. Each specimen was processed in duplicate. The initial PCR cycle employed a step-up program: beginning with a 5-minute denaturation at 95 °C, followed by 30 cycles at 95 °C for 40 s, 59.4 °C for 55 s, and 72 °C for 1 min, and concluding with a 7-minute final extension at 72 °C.

For the second PCR cycle, 2.5 µL of the amplicon from the first run was used as a template. This cycle utilized the same program but with internal primers to amplify a 298 bp fragment within the original sequence. It started with a 5-minute denaturation at 95 °C, followed by 30 cycles at 95 °C for 40 s, 65.7 °C for 50 s, and 72 °C for 55 s, ending with a 5-minute final extension at 72 °C. Positive and negative controls were included in each run of amplifications.

The PCR products were subjected to electrophoresis on a 1.5% agarose gel in 1× Tris-borate-EDTA buffer at 65 V for 1 h and were visualized under UV illumination to confirm the presence of DNA bands.

### Statistical analysis

Patient questionnaires and Nested-PCR results were analyzed. Data processing for descriptive statistics was conducted using SPSS software, version 26. Appropriate statistical tests, including the chi-square, Mann-Whitney U, and Kruskal-Wallis H tests, were selected based on the type of data. A *p*-value of less than 0.05 was deemed statistically significant. Crude odds ratios (ORs) with 95% confidence intervals (CIs) were calculated comparing MAP positivity in precancerous and cancerous groups versus controls.

## Results

### Demographic data

The results of our study, which examined demographic variables across three groups—patients with cancerous conditions, those with precancerous conditions, and HCs—revealed that the mean age of patients with cancer was 60.9 years. This was significantly higher than the control group’s mean age of 53.2 years (*p* = 0.003). A notable gender disparity was observed; women constituted 37.5% of the cancer group and 58.9% of the control group (*p* = 0.007). A history of cancer was reported in 35.7% of the cancer group, compared to 9.6% in the control group, which approached marginal significance (*p* = 0.07). The prevalence of hypertension showed no significant variation between the groups (*p* = 0.287). Drug abuse was significantly more prevalent in the cancer group (28.6%) compared to the control group (5.5%) (*p* = 0.013). No significant differences were found in bi-weekly dairy consumption (*p* = 0.082) or fast-food habits (*p* = 0.187) between the groups (Table 2). In our cohort, cancer patients were older and more often male than healthy controls. These differences in age and sex distribution are recognized risk factors for colorectal neoplasia and may partially confound the association between disease status and MAP detection.

**Table 2:** Demographic characteristics of the study participants

		Patients			Controls *n* = 73	*p*-value
		Cancerous *n* = 14	Precancerous *n* = 60	Total *n* = 74		
Age	Mean ± SD	60.9 ± 5.6	58.12 ± 7.5	59 9 ± 11.9	53.2 ± 11.5	0.003
Gender	Women	5 (37.5%)	22 (63.7%)	27 (36.5%)	43 (58.9%)	0.007
	Men	9 (64.3%)	38 (63.3%)	47 (63.5%)	30 (41.4%)	
History of Cancer	Yes	5 (35.7%)	10 (16.7%)	15 (20.3%)	7 (9.6%)	0.07
Hypertension History	Yes	4 (28.6%)	24 (40%)	28 (37.8%)	20 (27.4%)	0.287
Drug abuse	Yes	4 (28.6%)	4 (6.7%)	8 (10.8%)	4 (5.5%)	0.013
Bi-Weekly Dairy Consumption	Yes	0	10 (16.7%)	10 (13.5%)	18 (24.7%)	0.082
Bi-Weekly Fast-Food Habits	Yes	0	4 (6.7%)	4 (5.4%)	1 (1.4%)	0.187

^a ^SD; Standard Deviation

### MAP frequency by Nested-PCR assay

The results of this study revealed that the prevalence of MAP, detected using Nested-PCR, is significantly higher in precancerous and cancerous patients compared to HCs. Specifically, 46.7% of precancerous patients tested positive for MAP, a rate substantially greater than the 13.3% observed in healthy controls, with a *p* < 0.0001 denoting strong statistical significance. Among cancerous patients, the MAP positivity rate was even higher at 57.1%. Conversely, the negative rates for MAP were 35% in precancerous patients and 21.4% in cancerous patients. When considering the entire patient cohort, the MAP prevalence was 32.4%, again significantly higher than in healthy controls, as indicated by the same *p* < 0.0001 (Table 3). These findings suggest a potential association between the presence of MAP and the progression of these disease states, meriting further investigation. Although the numerical prevalence was higher in CRC than in precancerous lesions, our sample size did not permit a statistically robust comparison between these two patient subgroups.

**Table 3:** Detection of MAP IS900 sequence in precancerous and cancerous Patients, and healthy controls via Nested-PCR assay

		MAP prevalence by nested-PCR		OR	95% CI	*p*-value
		Positive	Negative			
Patients	Precancerous (*n* = 60)	28 (46.7%)	32 (53.3%)	5.51	2.38–12.74	< 0.0001
	Cancerous (*n* = 14)	8 (57.1%)	6 (42.9%)	8.40	2.40–29.36	
	All Patients (*n* = 74)	38 (51.4%)	36 (48.6%)	6.65	2.96–14.91	< 0.0001
Healthy Controls (*n* = 73)		10 (13.3%)	63 (86.7%)	Reference	-	

## Discussion

While MAP is a recognized pathogen causing disease in ruminants, its role in human health remains a subject of controversy [20]. First, the hypothesis linking MAP to CRC stems from its association with IBD like CD and UC, both of which elevate CRC risk [21]. Second, evidence of MAP detection in CRC patients’ intestinal tissue suggests its potential etiological role [22]. Third, high-magnification microscopy has revealed MAP organisms in both diffuse CRC and IBD-associated CRC, consistent with MAP’s ability to invade goblet cells—a process leading to acute and chronic hyperplasia, a known precursor to colon cancer [23]. The mechanisms underlying MAP persistence in the gut environment likely contribute to chronic inflammation and cellular changes that may drive cancer development [24]. Although the exact mechanisms remain unclear and warrant further investigation, MAP can also alter the host’s immune response, leading to persistent gut inflammation. This chronic inflammation may play a role in cellular changes conducive to cancer development. Furthermore, MAP’s presence could disrupt the balance of immune cells and cytokines, creating an environment favorable for carcinogenesis. By invading goblet cells, essential for mucus production and gut barrier integrity, MAP can induce both acute and chronic hyperplasia—a precursor to colon cancer [25–27].

The findings of the present study emphasize the significant relationship between the presence of MAP and the development of precancerous and cancerous conditions. Using Nested-PCR showed a higher prevalence of MAP in people with these conditions compared to healthy people, so that 46.7% of precancerous patients and 57.1% of cancer patients were positive for MAP. These rates are significantly higher than the 13.3% found in healthy subjects, and the statistical power of these results is strengthened by a *p* < 0.0001. The detection of MAP DNA in both early, precancerous lesions and invasive cancers suggests that MAP may be present throughout the neoplastic continuum; however, our study is underpowered to determine whether MAP prevalence truly increases with histologic progression.

Given the small size of the CRC subgroup (*n* = 14), conclusions pertaining specifically to invasive colorectal cancer must be interpreted with caution. In addition, the cross-sectional design precludes assessment of temporal order, raising the possibility that MAP colonization occurs as a consequence of neoplastic mucosal changes rather than preceding them, thereby highlighting reverse causation as a plausible alternative explanation.

Although we observed a robust statistical association between MAP DNA detection and colorectal neoplasia, our case-control, cross-sectional design cannot determine the temporal sequence of events. It remains unclear whether MAP contributes to the initiation or promotion of neoplastic lesions, or whether the altered mucosal environment of adenomas and carcinomas simply provides a niche that favors MAP colonization. Tumor-associated changes in local immunity, mucus composition, or epithelial integrity could plausibly facilitate opportunistic colonization by environmental organisms such as MAP. Thus, our findings demonstrate correlation rather than causation. Longitudinal studies assessing MAP status before the development of colorectal lesions, as well as mechanistic experimental models, will be essential to determine whether MAP presence precedes and contributes to tumorigenesis or instead represents a secondary phenomenon.

In 2017, Zamani et al. [12] presented evidence that a high prevalence of MAP DNA and anti-MAP antibodies in Iranian patients with CD is significantly associated with the development of CD. Despite the involvement of various factors in IBD, the presence of MAP DNA and antibodies in Iranian CD patients suggests a potential transmission of MAP from animal-derived products to humans. In 2018, Ellen S. Pierce [23] proposed that MAP could be a contributing factor to CRC, particularly in the context of inflammatory bowel diseases like CD and UC. Additionally, in 2023, Mintz et al. [28] suggested that MAP might infect specific cells, leading to vessel blockage, new vessel growth, and the characteristic “creeping fat” observed in CD. There’s a hypothesis that MAP may be more prevalent in the mesentery (the tissue that connects the intestines to the abdominal wall) rather than the bowel wall, potentially influencing Crohn’s pathology. Confirming the presence of MAP in large numbers within Crohn’s tissues could help establish its role in the disease.

The potential association between MAP and CRC carries important public health implications, particularly in regions where MAP infection is common in livestock. This underscores the need for rigorous investigation into MAP’s role in human disease and the extent of its public health impact. It also highlights the importance of considering zoonotic and environmental pathogens in CRC etiology within the broader One Health framework.

This study has several limitations. First, as a single-center case–control study with a cross-sectional design, it can only demonstrate an association between MAP DNA detection and colorectal neoplasia and cannot establish temporality or prove causation. Second, patients and controls were not fully matched for age and sex, and our sample size—especially for CRC—was modest, limiting the robustness of subgroup analyses and precluding stable multivariable adjustment; residual confounding by demographic and lifestyle factors is therefore likely. Third, we relied on nested-PCR for detection of MAP DNA in mucosal samples. This highly sensitive method cannot distinguish viable from non-viable organisms, we did not confirm positives using a second target or sequencing, and sampling error may have led to false negatives. Fourth, we did not measure inflammatory markers, immune responses, or molecular signatures that could elucidate mechanisms linking MAP to tumorigenesis, nor did we fully explore potential interactions with diet, substance use, or other lifestyle factors collected in our questionnaire. Finally, our participants were all from a single province in Iran, where environmental exposure to MAP from livestock may differ from other regions, limiting the generalizability of our findings. These limitations underscore that our results should be viewed as preliminary and hypothesis-generating.

## Conclusions

While the association between MAP and CRC is compelling, it is still inconclusive. Further research is necessary to clarify the relationship and explore the implications for the diagnosis, treatment, and prevention of CRC. Understanding the role of infectious agents, such as MAP, in the development of cancer can open new ways to fight this disease.

## Data Availability

The datasets used and/or analysed during the current study available from the corresponding author on reasonable request.
